# Supplementary material for: Promoter-Bound Full-Length Intronic Circular RNAs-RNA Polymerase II Complexes Regulate Gene Expression in the Human Parasite Entamoeba histolytica
Source: Noncoding RNA. 2022 Jan 27;8(1):12. doi: 10.3390/ncrna8010012 (PMC8876499; doi:10.3390/ncrna8010012)
Supplement: Supplementary file 1 [file ncrna-08-00012-s001.zip › ncrna-1458591-supplementary/suppl mat upload/FS1-FS5.pdf]

Supplementary Figures

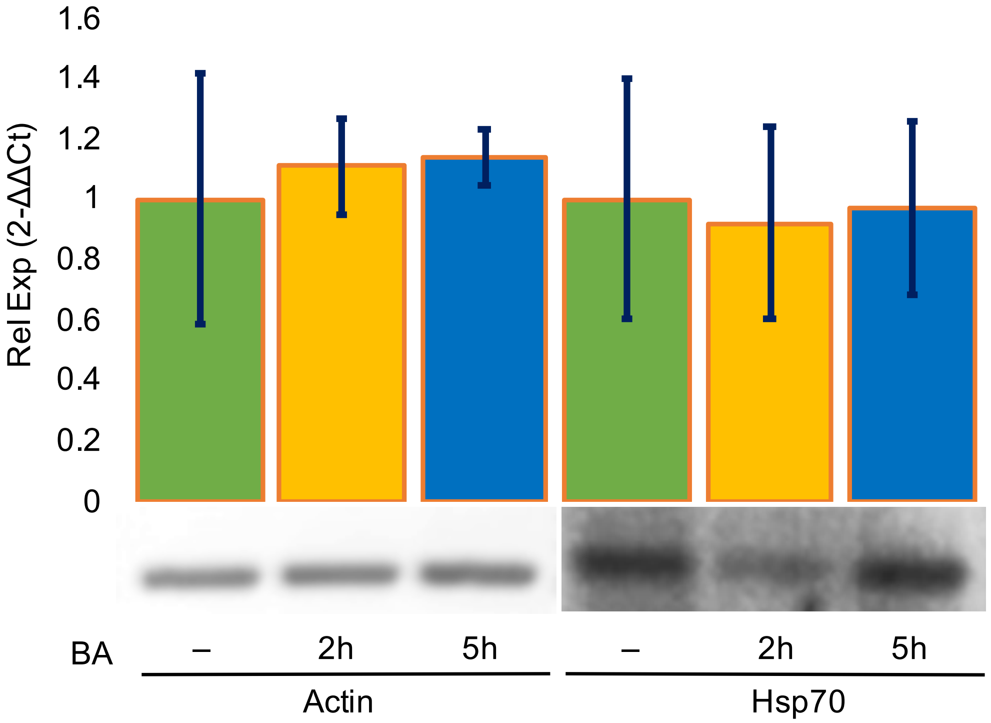

Figure S1. Boric Acid treatments do not affect the transcription of housekeeping genes. *E. histolytica* trophozoites were incubated in 5 mM H_3BO_3 for 2 or 5 h. RNA was extracted, and Actin and Hsp70 were amplified, visualized, and analyzed by qRT-PCR. Relative expression is compared to one in the HA control without BA. Expression profiles are statistically non-significant (ANOVA and Turkey test *a posteriori*). Standard deviations are shown as thin blue bars. Representative electrophoretic images of PRC products are shown.

Figure S2. Identification of TIA-1 binding motifs on flicRNA sequences from *Entamoeba histolytica*.

Selected homolog motifs: TIA1(Hs/Mm): uuuuubk, TIA1(Hs/Mm): uuuuu**.**

Stringency level: High**, c**onservation filter: On

(Paz I, Kosti I, Ares M, Jr., Cline M, Mandel-Gutfreund Y. RBPmap: a web server for mapping binding sites of RNA-binding proteins. Nucleic acids research. 2014;42(Web Server issue):W361-7. Epub 2014/05/16. DOI: 10.1093/nar/gku406. PubMed PMID: 24829458; PubMed Central PMCID: PMCPMC4086114)

flicX13

==

**Sequence Position**	**Motif**	**K-mer**	**Z-score**	**P-value**
**27**	**uuuuu**	**uuuua**	**2.523**	**5.82e-03 ***
**61**	**uuuuubk**	**auuugu**	**2.598**	**4.69e-03 ***
**111**	**uuuuu**	**uuuua**	**1.947**	**2.58e-02 ***

flic670i1

==

**Sequence Position**	**Motif**	**K-mer**	**Z-score**	**P-value**
**70**	**uuuuu**	**uuuua**	**2.561**	**5.22e-03 ***

flic670i2

==

Sequence Position	Motif	K-mer	Z-score	P-value
83	uuuuu	uuuua	2.606	4.58e-03 *
148	uuuuubk	auuuuuu	3.422	3.11e-04 *
149	uuuuubk	uuuuuuu	3.735	9.39e-05 *
150	uuuuubk	uuuuuug	3.843	6.08e-05 *
151	uuuuubk	uuuuugu	3.539	2.01e-04 *

flic510

==

Sequence Position	Motif	K-mer	Z-score	P-value
77	uuuuu	uuuua	1.917	2.76e-02 *
179	uuuuu	uuuuu	3.227	6.25e-04 *
180	uuuuu	uuuuu	3.227	6.25e-04 *
181	uuuuu	uuuua	3.061	1.10e-03 *
202	uuuuu	uuuua	3.061	1.10e-03 *

flic170

==

Sequence Position	Motif	K-mer	Z-score	P-value
26	uuuuu	uuuuu	2.947	1.60e-03 *
50	uuuuubk	auuuucu	2.667	3.83e-03 *

**117**	**uuuuubk**	**auuuuug**	**3.206**	**6.73e-04 ***
**118**	**uuuuubk**	**uuuuugu**	**3.176**	**7.47e-04 ***
**181**	**uuuuu**	**uuuuu**	**2.871**	**2.05e-03 ***
**182**	**uuuuu**	**uuuua**	**2.758**	**2.91e-03 ***

Supplementary Figure S3.

Identification of promoter elements in *Entamoeba histolytica* genes.

Inr: Initiator

RabX13 EHI_065790

Pr-RabX13 F

TAATGTTTTATTCAGATGAAGTTCAAAGTTTTCTTTTTTTTTATTATTATTTTTAGTTTTTTTTTAAGATTTT

TATA box — Inr

TATTTTTAGTTTTTTTTT**TATTTGAAA**AA**AAAAATTAA**ATTAATTTTAAGTTCTGTTCTTAACGAA**ATG**GCACA

Pr-RabX13 R

ACATGTAAGAAATACTAACCGTTTAACTCCTTTAAAATTAATCGTTGTTGGAGACTCTTCAGTTGGAAAAACA

EHI_169670

Pr-169670 F — TATA box

AAATGAAGATAAAATTAAAATAAAATTGAAATACCATTTATAGTAACATTAATATATTTAAAGTACTTAATAGTTTACAATACTCTGTTT

GAAC box — GAAC box — Pr-169670 1 R

TTAGTCTTTTTTAATTTTCTGAACTTTACTATATAAAGAGAACTCAATATTATTAGGTTTCAA**ATG**AAGTCATTACTTAAGAAGAATATT

EHI_014170

Pr-014170A F

CAACAATGTATATTGCTCCAACCAATGACTTTAGTGCTATTGATTCTTTATATAACCTTAAACCAACTTC

AGAACAACCTCATCATCTACGCTGTTTAAGAAAATCACTATTCATTTCAAAACAACAACACTGTCACTCA

Pr-014170B F

GAAC box

GTCCATATTGTTTCATTTATTACTTCTGAAGAAGCTTTATCTGATGTAGTTTTAAGACAAAA**GAACTTC**

AAACATTACGTAGTTATCACGTTCAATTTGTTTTATTAGGAGATGTATTAAATCCATTAAGCCATACCCG

Pr-014170A R

TTCCTTAGTTCGTTCTTTGGTTAAACGACAAAGTCGTGGATGGGGAGTAACTGAAAACGATTTAACAAAA

ACAAATCCTTATGAGCGTTTAATGATGAGAATGTCTGAAGGCATTATCATTTTGCCTCGTCGTAAAGTAT

Pr-014170D F

TAAAACAATTACCTCATATGAGAGCTGGACAAGTTCCGTGTGAAAGTAAAGCTGGCAAATTTATTTCATG

TAAAGTAATTACACATATGACAAATGATACAGTTTCAATGATTCCAGTACCAATTATTTCTAAACAAAAG

Pr-014170B R

AATTTAAGCACTATCAGATGTGGTAAATCAAATATAGTTGATGGAAAATGTGTGTCTTCAGAAGAACGAG

TATA box

GAATGCTTTTCTTGGTTGAATTATCTCCGATTT**TATTTAAA**TTGATTTGGAAAACCGAAAATGAGATAGA

Pr-014170E F

CCAT**TTGACA**ATATTTTATGGTGAGACAAGTTGTAAGCAGATAAAAAAAGGTAAATATAACTTCTTAATT

Pr-014170D R

TTTGAAGGTAATAAACCAAATGGTAGTCTTACTAAAACAATTTATTGGACTACTTTGGATACAAAAGAAT

TTTGTACGGTTTTTGAAAAAGCTATGAGTCAAGATGTTCCACAAAATTATATAAAAATTATGCAAACTAT

TTGGAATGATAATAAGAAGACATTAAGTAGTATGGAAGAAAAATATATGAGTATGAAGAGGGAAAAAGAA

Pr-014170F F

Pr-014170E R

ATTGTGAACAAAATGGATGAATATGTAAAAGGTGTTCCTAAAACAGAACTAAATAGAAAAGTAAGAAATG

AAGCTGATAAAAAAACAGAACCAAAAGAGCCAAGTACTAATTAATAAATAAAATGTCCAAATTTTTTGCT

AATCATTCACGTACATTTATTTTTTTGTTATTTAATAACTGGTTAATTTTGTATTAGGAAAAGAATAATA

Pr-014170G F

AAATAAGCACTTAATTAAATGTTAATAAACTCTATTTTTTTTATTCCTTGTTCAAGTGAGACAAATCCTG

TCTTTTTATCTTATTTTTTTTTATTAGGAAAAGAAAAAGAGAAAAAAATAACAAAATAAAATAATAAAAAT

TATA box

TTTATTAAAAATGTAATTTTTATTTTGAAGAAAGGAAAAGAAAGTCAAAAATTTTGTTAAATAAAATATA

Pr-014170F R

AATGAAACGAATACGATTAAAAGAAGTTAAGGAATGTTGAGTCATACTAACGTAATACAGAAAAGAAGAA

Inr

GAGATCTTGAAATTAAAAAAGGAATAAAGAATGAAATCATTATTAAAGAAGAATATTAAACAAGACTATA

Pr-014170G R

CCATTGAATTTAAACATATTAAAGGACTAGATATTGGAGACAATATTGAAGTTGTTTTAAAATGGAGAAG

EHI_192510

Pr-192510A F

GAAC box

AAAATGGTTACGCTTGAATGAGGGGTGATATGAACTTTCTCGCAAATATTTCCATGACGACAAGATCCAA

Pr-192510B F

CTTTAGAGAAGAACTCACAGAGATTGAGACTTTCCATCAATAACTAAAAACTATTTTTCGTTTTGACAAT

TATA box

TTTTAATTTTTTAATTTTTTTATCAGAGTTTTAAATTATTAATTTTTTAAAGAATGATGTCTCAGTTTTT

Inr Inr

TTAATAAAAGTGAAACATCTGCTAAAAAAATTAAGATAAATTTCATTTAATAATGTCTTCTCAAAAAATA

Pr-192510A R Pr-192510B

GTTAGTGTTGGAATTGATTTAGGAACTACTTGTTGTTATGTTGCTTGTATTGAAGATGGTAATGAACCAA

AAATACTTCTAATGAATAATGAAGACTATTTACCTTCATGGATTTCTCTTTCAAATGTTAAGAATGGTTT

EhRNF EHI_098590

Pr-098590 F

AATTAAACAAGATTCTCCAGGTGTTGATTGGTCAATTTCATTCTATTCTGACAAAGAAAAAGATCCTTCA

TATA box

TTTTATAATGTTTATCAGTGTTTTACATTACGTCCACCTTTAAACCAACACAACTCATCATTAAATCCTG

AATTTAAATTTGAGTATGAAAATGCTATTGATGTTAGTGCGTTTACTGGAAATAAAGAAAACTTTGCAGT

AAAACGATTATTTTTCCTTCAATGGTATCAATAAAACTTTGTAATTTTTTATTCACTTTTTCTTATTATT

Inr GAAC box

TTGTTAAAAATTCTGTTGAATAATAAAAGGGGAAAAAAAGAACTTATTTTTTTTATTTCTTTATCTTTTT

Pr-098590 R

CTTATGGGTGCTGAACCTTCAAAACCAGTTATTTCTAATGATGTTAATAGAATGTGTTCAACTATTACAG

EhNudC EHI_023890

TATTAAATCATCCAAACATTAAGAATTTGCCAAAAGCAATTATTTGTGCAGAGGAGACTTTGTTTTGGAGATACCAAAATA

P-nudc S 500

TAAGTAATACTAACTTATCAACTATTGAGGCTATCTATTATTTTTATAGAGATTATTATACTTGTATGAATGGTAAATATA

ATGGAGAATGTGATGATCTTTTATATTTCTTCACCCATATTTATCATCGTGTCCAAGACTTTTATAACGAACATCCAGATA

AAACGTTCATCCACAAAGATGGATATATTCAATACAAAACACCAGAGCTTGAAAAACAAAAAGAAGAAGAATACAAAAAGA

P-nudc S 250

GGGAAGAGATGATTGAATCTAAAAAACAATCTAAAGCACAAAAAAATAATTAAATATTTTTGTTATTTAAATGGTTTGCAT

P-nudc AS 250

TACTCTTATAATTTTTTTTAAAGAACAAAAAGAATGAAGAAAGATTAAATGTTGTTTCGTATTAATAATCTCAATCACAAA

GAAC box

TGAACAAGTTGGTACTAAAAAATCTAAAAAGAAAAAGGATGATCATCACCATGTCAGTCCACGTCATTCATTCGTTGAAAG

TATA box Inr P-nudc As1

ATAATATTTAAAGATAGAACTTAGACAAAAAAACAAAATCGTTGCGTTATGGCTGGATGGAAACCATCTCCTTATGTTAAA

TATGGATATACCGAAAAGAAGTATAAATGGACTCAGACACCTGAAGATGTAACATTAACAATAGAATTACCTGATGAAAAC

TTAAAAACAAAAACAGATATTCAAATAATAATGAAAACAAATAAATTTGAAATGATTATTAAAGGAGAAGTTTATATTGGA

GGAGAATTAGAACATGCTATTAATGTAGATGATTCTACATGGACT

EhSMC EHI_187190

TAAACAAAATGAAAAATTGTCCCCAATAATTTAATTAAAAGAGAATGCCATGAATGAAAAATGATCCAATAAATAACGTTA

TTCCAATTATATAAATAATTGCAGTTTCTACTTATAAATTATAATTCAATCAATTAAAATAAAATATACAAACTTTTATCT

GACATTGTTGTAAAATCAGGAAATCCTAAGTAATTTGCAACTGCATGGCATAAAATGCATGAAATTACAGATTTTGTACTG

AAGAAAACATATGACGAATACATTCCAAATAATGTAGTAAATCCTACTTGTATCATTGTATTGATGATAGCAGCTTTATCA

ATAACATCTTCTGTTATTAGGTGGTTGATGTGTGCAAGTCCAAAAAGCATTGAACAATAGATAAATGTAAATATTGATGTG

P-smc S 500

CATCCGTTTGTCAACAGGTATGGTATAAACACTCCTCTAAATAATGTTTCTTCAATTATTGGTGCAACAACAAGTGCCCGT

ATGATGAAAAGTGAATTAGGACAATATATTTGATCAGTTTGTTGATAAACACAATATATAAATCCAATAAAAAGAATGACG

ATTTGGGTTAGATGAAATAACGTTGATAAAACAGGAATATTAAAGTTAAGAGGGACATCAAGAATAATAGTGACAAATACA

P-smc S 250

CAATATCCTATTCCAATGATCATTCTTATTATTGCTATTTTATTTTCGTCATTTATTCTATGTGAAGCTATACCCAAAAAA

P-smc AS 250 TATA box

GCAAGAAGAATGACTGTCAATGTTAATGTTATGATAATACTCATAAACCATCCAACAACTATCATTTAAACTATAAATTAT

GTATTTGTTTTTTTTTAATTTCCAAATAAAGTGTATAGTAATAAATATAGTAAATTTGCTATAATTTACTACTATAAAACT

Inr P-smc As1

ATATAATAAAAATTGAAAATAAACCATCATTACATGGATAAGACTCAATCTGATGTGCTTGCTCACTTCCTTGAACAGGAA

CTAAGTGTTGACCAAAAAGTAAAGAGGATACAAAGAAAGGTAGCAGAGTATATGAAAAACTATAATTATGATGAATATGTA

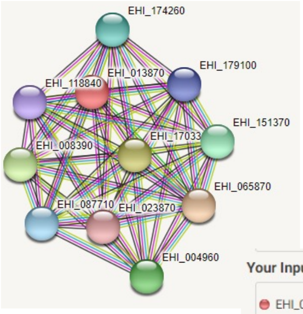

EHI_013870 (METTL3 ortholog): rRNA adenine N(6)-methyltransferase; Dimethyladenosine transferase

Your Input:

EHI_013870 *rRNA adenine N(6)-methyltransferase; Dimethyladenosine transferase, putative ; Belongs to the class I-like SAM-binding methyltransferase superfamily. rRNA adenine N(6)-methyltransferase family (295 aa)*

Predicted Functional Partners:

Protein	Description	Neighborhood	Gene Fusion	Cooccurence	Coexpression	Experiments	Databases	Textmining	[Homology]	Score
EHI_065870	*Rna-binding protein pno1; RNA-binding protein, putative*	●			●	●		●		0.996
EHI_170330	*RIO1 family protein*				●	●		●		0.996
EHI_008390	*Pre-rrna-processing protein tsr1; Uncharacterized protein*				●	●		●		0.995
EHI_004960	*Essential nuclear protein 1; Bystin, putative*				●	●		●		0.994
EHI_151370	*Periodic tryptophan protein 2; WD domain containing protein*				●	●		●		0.994
EHI_174260	*Hypothetical protein; Uncharacterized protein*				●	●		●		0.994
EHI_087710	*Rna-binding protein nob1; Belongs to the NOB1 family*				●	●		●		0.991
EHI_179100	*U3 small nucleolar RNA-associated protein 22; Uncharacterized protein*				●	●		●		0.989
EHI_118840	*Rrna 2'-o-methyltransferase fibrillarin; Fibrillarin, putative*				●	●		●		0.988
EHI_023870	*U3 small nucleolar RNA-associated protein 4; WD domain containing protein*				●	●				0.982

Your Current Organism:

Entamoeba histolytica

NCBI taxonomy Id: 5759
Other names: E. histolytica

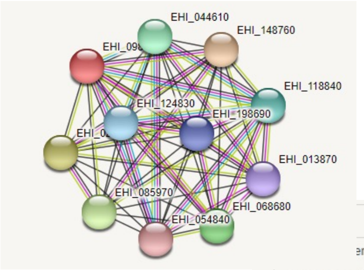

EHI_098730: Trna (cytidine32/guanosine34-2'-o)-methyltransferase; Ribosomal RNA methyltransferase

...ent protein-protein associations	Known Interactions	Predicted Interactions	Others
associations are meant to be specific and meaningful, i.e. proteins jointly contribute to a shared function; this does not necessarily mean they are physically binding to each other.	*from curated databases*<br>*experimentally determined*	*gene neighborhood*<br>*gene fusions*<br>*gene co-occurrence*	*textmining*<br>*co-expression*<br>*protein homology*

Your Input:

EHI_098730 *Trna (cytidine32/guanosine34-2'-o)-methyltransferase; Ribosomal RNA methyltransferase, putative (231 aa)*

Predicted Functional Partners:

Protein	Description	Neighborhood	Gene Fusion	Cooccurence	Coexpression	Experiments	Databases	Textmining	[Homology]	Score
EHI_148760	*Trna (guanine26-n2/guanine27-n2)-dimethyltransferase; N2,N2-dimethylguanosine tRNA methyltransferase, putative*				●	●		●		0.988
EHI_023130	*Trna (guanine10-n2)-methyltransferase; Uncharacterized protein*				●			●		0.987
EHI_085970	*Trna (adenine58-n1)-methyltransferase non-catalytic subunit; Uncharacterized protein*				●			●		0.986
EHI_068680	*Nucleolar phosphoprotein Nopp34, putative*				●	●		●		0.979
EHI_044610	*Rrna small subunit pseudouridine methyltransferase nep1; Ribosome biogenesis protein NEP1, putative*				●		●	●		0.976
EHI_118840	*Rrna 2'-o-methyltransferase fibrillarin; Fibrillarin, putative*				●	●	●	●		0.976
EHI_124830	*Hypothetical protein; Uncharacterized protein*				●	●		●		0.974
EHI_198690	*MAK16 protein, putative ; Belongs to the MAK16 family*				●	●		●		0.971
EHI_013870	*rRNA adenine N(6)-methyltransferase; Dimethyladenosine transferase, putative ; Belongs to the class I-like SAM-binding m...*				●	●		●		0.965
EHI_054840	*U3 small nucleolar RNA-associated protein 5; Uncharacterized protein*				●	●	●			0.962

Figure S4. String analysis of loci EHI_013870 and EHI_098730. Protein products of these loci were identified as members of pre-mRNA ribonucleoparticles [Valdés *et al*., Proteomic analysis of *Entamoeba histolytica in vivo* assembled pre-mRNA splicing complexes. J Proteomics. 2014]. Whereas only locus EHI_013870 codes for the METTL ortholog involved in adenine N(6)-methyltransferase, locus EHI_098730 codes for a tRNA (cytidine32/guanosine34-2'-o)-methyltransferase, but its predicted protein-protein interactions show association with the EhMETTL ortholog.

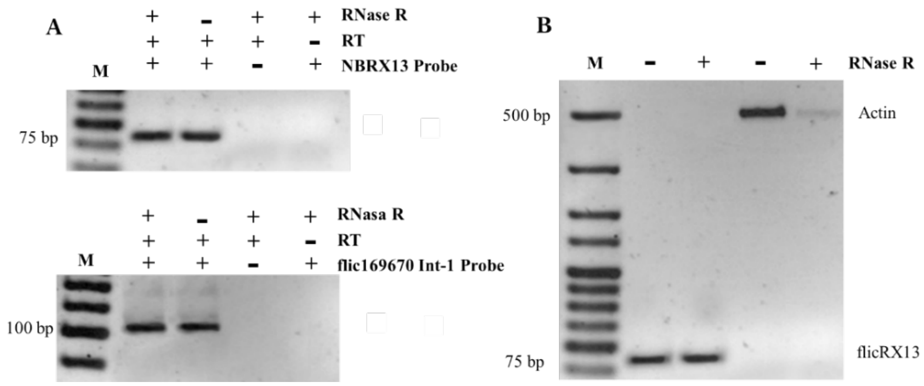

Figure S5. Validation of flicRNAs with RNase R treatment. (**A**) Primers that span the 5'-3'ss junctions were used to identification flicRX13 and flic670i1 by divergent RT-PCR with purified RNA treated with RNase R. Amplicons were resolved in 2.5% agarose gel. (**B**) Analysis of linear RNA degradation efficiency by exoribonuclease R. Amplification of the constitutive transcript Actin versus flicRX13 from treated RNA was compared. M, molecular weight marker.
