# Supplementary material for: Promoter-Bound Full-Length Intronic Circular RNAs-RNA Polymerase II Complexes Regulate Gene Expression in the Human Parasite Entamoeba histolytica
Source: Noncoding RNA. 2022 Jan 27;8(1):12. doi: 10.3390/ncrna8010012 (PMC8876499; doi:10.3390/ncrna8010012)
Supplement: Supplementary file 1 [file ncrna-08-00012-s001.zip › ncrna-1458591-supplementary/suppl mat upload/GL Table S1.pdf]

Table S1. *Entamoeba invadens* orthologs and paralogs of *E. histolytica* virulence-related genes; p, *E. histolytica* paralogs. Stage conversion transcriptomic data is shown. Shaded in green appear the loci in which circRNAs have been identified.

E. histolytica	*E. Invadens*	Paralogs		
EHI_169670 (p)	EIN_391640	EIN_186580		
EHI_014170 (p)	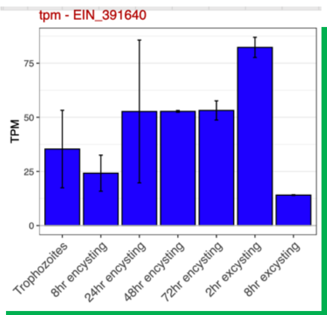<br>EIN_391640	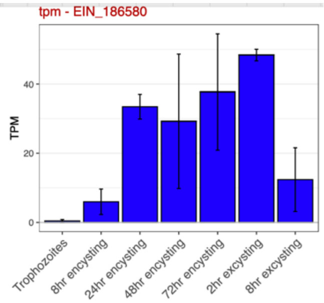<br>EIN_186580	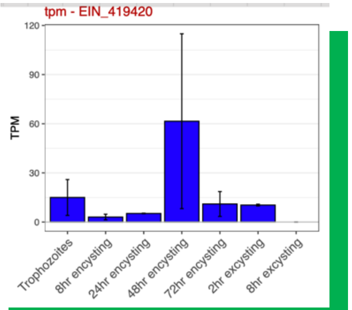<br>EIN_419420	
EHI_042870	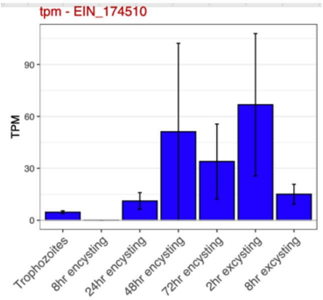<br>EIN_174510	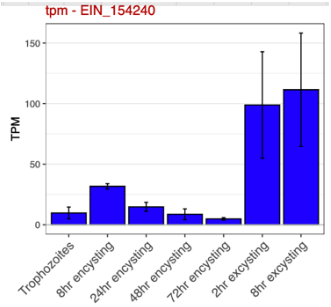<br>EIN_154240		

<table>
<tr>
<td>EHI_083590</td>
<td>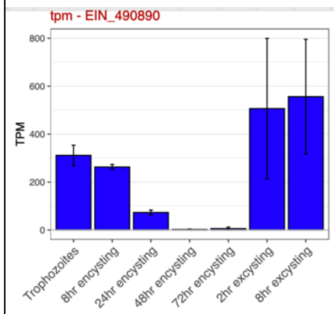

EIN_490890</td>
<td></td>
<td></td>
<td></td>
</tr>
<tr>
<td>EHI_192510</td>
<td>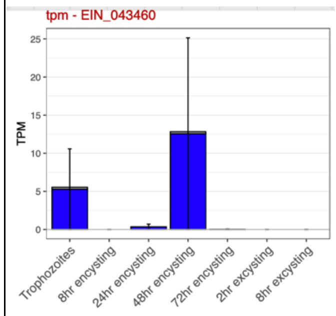

EIN_043460</td>
<td>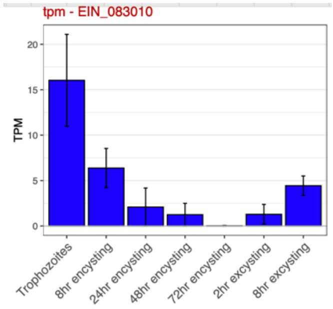

EIN_083010</td>
<td>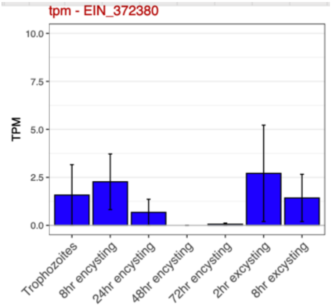

EIN_372380</td>
<td>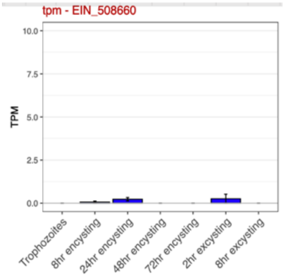

EIN_508660</td>
</tr>
</table>
