# Supplementary material for: Promoter-Bound Full-Length Intronic Circular RNAs-RNA Polymerase II Complexes Regulate Gene Expression in the Human Parasite Entamoeba histolytica
Source: Noncoding RNA. 2022 Jan 27;8(1):12. doi: 10.3390/ncrna8010012 (PMC8876499; doi:10.3390/ncrna8010012)
Supplement: Supplementary file 1 [file ncrna-08-00012-s001.zip › ncrna-1458591-supplementary/suppl mat upload/GL Table S2.pdf]

Table S2. Genes (Gene Accession), oligonucleotides and conditions are described in the main text. Amplifications were done at 35 and 38 cycles. US, unspliced; S, spliced variantes, respectively.

Genes	Oligonucleotides		PCR conditions
	**Name**	**Sequence (5' ➔ 3')**	
RabX13 (EHI_065790)	Rab2 F	CGTTGTTGGAGACTCTTCAGTTGG	94°C,45'', 60°C,45'', 72°C,45''
	Rab2 R	GACCCATTTCAGTTGAAACAGTTC	
	Rab2BSs	GATGAGATGAGATAAGATTTTATC	94°,45''; 55°C,45''; 72°C,30''
	Rab2BSas	CCGAATTACTGTCTTGAAAATC	
	NBRX13	CTTTTAAAGTATTTAAAACAAACCTAAATAAAAGTTAGTTTATATTTATT	94°C,45''; 55°C,45''; 72°C,30''
	Pr-RabX13 F	TAATGTTTTATTCAGATGAAGTTC	94°C,45''; 58°C,45''; 72°C,45''
	Pr-RabX13 R	TTT CCA ACT GAA GAG TCT C	
	Rab2Rex 2	TTGAACACCACGATAATAACTAGT	
rpL12 (EHI_191750)	L12BSs	GATACTTTATAACAATATTTTGGTGG	94°C,45''; 56°C,45''; 72°C,30''
	L12BSas	GAATACTTTAATAACGTATGGATG	
rpS14 (EHI_074090)	S14BSs	TTAACAAATGAAGTTGTTTTAAC	94°C,45''; 51°C,45''; 72°C,30''
	S14BSas	AATGAAATGTGTAGTGTAACAAAC	
Clc2B (EHI_186860)	Cdc2BSas	ATTTATTTTTATTTCATTTTATATT	94°C,45''; 47°C,45''; 72°C,30''
	CloBSs	GAAAATCTATGTTTATAAAAAC	
	CloBSas	CCAACAAAAATAAAATAACAAAC	
	Pr-CL2B A F	TTACTAAACACAAGATAGAATAG	94°C,45''; 51°C,45''; 72°C,45''
	Pr-CL2B A R	AAACTTGTCTTAGAGATAGTC	
	Pr-CL2B B F	ATATGTTTTAGTATTTCCTG	94°C,45''; 46.5°C,45''; 72°C,45''
	Pr-CL2B B R	TATATTTACTAGTTCTATTAATG	
EHI_169670	circRev169670	GGCAAGAGAATTGATTGATTAAAG	94°C,45''; 55°C,45''; 72°C,30''
	circF169670	CTATTATCTACACACACTCATTTC	
	circProbe Intron 1	GGAGTATATTCAAAATTAAACAAACCTATAAATGATTAGTG	
	circINT2Rev169670	CAAATATTCTCCCCTCAACAACTC	94°C,45''; 55°C,45''; 72°C,30''
	circINT2F169670	GAGTAAGTTAGTTCTTTAG	
	circProbe Intron 2	CATATATATAATAAAAAAACAAACCTACAAATAAG	
	EHI169670_E2s	AAGAAAGTTAATGATTCTGAGAAAGAG	94°C,45''; 58°C,45''; 72°C,45''
	EHI169670_E2as	CTTCTTTTTCTTTTTCTAATTCTTCACCC	
	Pr-169670 F	TACCATTTATAGTAACATT	94°C,45''; 56°C,45''; 72°C,45''
	Pr-169670 1 R	CATTTGAAACCTAATAATATTGA	
	Pr-169670 ExU F	AAGATAAAGAAATAGAAGATGCAGTTAAAG	95°C,50''; 52°C,45''; 72°C,50''. Melt 52°C,0.5''
	Pr-169670 ExU R	CTTCAGCAGCAACTGAAATTG	
	circRev192510	CATATGCTTCTTCATTTCTTC	94°C,45''; 55°C,45'';
	circF192510	GAGAAAGGAATGAGCCAAC	

	circProbe 192510	CCATTTCTACTCTCATCTCCCCTATTATTTCTACCTTGAAATATGC	72°C,30''
EHI_192510	Pr-192510A F	AAATCAATTCCAACACTAAC	94°C,45''; 56°C,45''; 72°C,45''
	Pr-192510A R	TTGAGACTTTCCATCAATAAC	
	Pr-192510B F	ATTACCATCTTCAATACAAGC	94°C,45''; 58°C,45''; 72°C,45''
	Pr-192510B R	TGACTTTAGTGCTATTGATTC	
	Pr-192510 ExU F	TATATCAAGATGGGAAAAACTACAAAGATA	95°C,50''; 52°C,45''; 72°C,50''. Melt 52°C,0.5''
	Pr-192510 ExU R	TGGCTTTGTCACTGTTGCTT	
EHI_014170	circRev014170	GAGAGTGTAGAAGGAGAG	94°C,45''; 55°C,45''; 72°C,30''
	circF014170	GTTGGAAATACTCATTTTTATTAG	
	circProbe 014170	GAAGTGGTGATGGAGAAATAAAAACAAACCTTATTTAATTC	
	Pr-014170A F	TGACTTTAGTGCTATTGATTC	94°C,45''; 54°C,45''; 72°C,45''
	Pr-014170A R	TAACCAAAGAACGAACTAAG	
	Pr-014170B F	TCACTCAGTCCATATTGTTTC	94°C,45''; 54°C,45''; 72°C,45''
	Pr-014170B R	ATTTACCACATCTGATAGTGC	
	Pr-014170D F	ATGAGAGCTGGACAAGTTC	94°C,45''; 56°C,45''; 72°C,45''
	Pr-014170D R	TAAGACTACCATTTGGTTTATTACC	
	Pr-014170E F	TATGGTGAGACAAGTTGTAAG	94°C,45''; 54°C,45''; 72°C,45''
	Pr-014170E R	TCTATTTAGTTCTGTTTTAGGAAC	
	Pr-014170F F	ATTGTGAACAAAATGGATG	94°C,45''; 51°C,45''; 72°C,45''
	Pr-014170F R	TGTATTACGTTAGTATGACTC	
	Pr-014170G F	TATTCCTTGTTCAAGTGAG	94°C,45''; 51°C,45''; 72°C,45''
	Pr-014170G R	TATTGTCTCCAATATCTAGTC	
	Pr-014170 ExU F	TAACTCAACAAAAGAAAATTTCTTCTTTAG	95°C,50''; 50°C,45''; 72°C,50''. Melt 52°C,0.5''
	Pr-014170 ExU R	TTTCTTTCTTTAATTCTTCAAGT	
EHI_042870	Pr-042870 F	TTTCATTCATAAAACAAGAAG	94°C,45''; 49°C,45''; 72°C,45''
	Pr-042870 R	AAATACATTGATGTCCAAG	
EHI_083590	Pr-083590A F	TTGATACATTTGATGGTCCTG	94°C,45''; 55°C,45''; 72°C,45''
	Pr-083590A R	AATAAAATAAAACTGATGGTGAGG	
	Pr-083590B F	TATTAGTTCTCAACTTTAATCACTTC	94°C,45''; 55°C,45''; 72°C,45''
	Pr-083590B R	TTTGAGGCTCTAACGAATTTGC	
EhRNF (EHI_098590)	Pr-098590F	TAAACAAGATTCTCCAGGTG	94°C,45''; 54°C,45''; 72°C,45''
	Pr-098590 R	TTCTATTAACATCATTAGAAATAACTG	
	EHI_098590ExU F	CCAGACTTTTTGATGTTAGAACAAAAACG	95°C,50''; 52°C,45''; 72°C,50''. Melt 52°C,0.5''
	EHI_098590ExU R	TTGGAGGTGGGGTATATCGT	
EhNudC (EHI_023890)	P-nudc As1	AACGCAACGATTTTGTTTTT	95°C,50''; 45°C,45''; 72°C,45''
	P-nudc S 250	TAAAGCACAAAAAAATAATT	
	P-nudc AS 250	AATTATTTTTTTGTGCTTTA	95°C,50''; 45°C,45''; 72°C,45''
	P-nudc S 500	CTATTATTTTTATAGAGATT	
	Pr-023890 F	GAAGATGTAACATTAACAATA	95°C,50''; 50°C,45''; 72°C,50''. Melt 52°C,0.5''
	Pr-023890 R	GACTTCAATTATTTTTCCAT	
	P-smc As1	GTAATGATGGTTTATTTTCA	95°C,50''; 45°C,45''; 72°C,45''
	P-smc S 250	TGATCATTCTTATTATTGCT	

EhSMC (EHI_187190)	P-smc AS 250	AGCAATAATAAGAATGATCA	95°C,50''; 45°C,45''; 72°C,45''
	P-smc S 500	ATGGTATAAACACTCCTCTA	
	Pr-187190 F	GGATAAGACTCAATCTGAT	95°C,50''; 48°C,45''; 72°C,50''. Melt 52°C,0.5''
	Pr-187190 R	CATTCTATCTAAAACTCCTT	
Eh actin (EHI_107290)	EhActF	GAGCTGTATTCCCATCCATTGTTG	94°C,45'', 65°C,45'', 72°C,45''
	EhActR	CTTTCAGCAGTAGTGGTGAAAGC	
EhRNA polymerase II (EHI_121760)	Pol II F Xma I	ATCCCCCGGGATGACTGAACTCAACATGGAAACGAAAC	94°C,45'', 58.5 °C,30'', 72 °C,45'
	Pol II R Xho I	GCAACTCGAGTCATTTATCTTGCGGTTTAGGGAATATACTAGG	
EhRNA polymerase II (EHI_056690)	RNAPIIs	GATCCAACATATCCTAAAACAACA	94°C,45'', 60 °C,45'', 72 °C,45'
	RNAPIIas	TCAATTATTTTCTGACCCGTCTTC	
HSP70 (EHI_052860)	HSP70s	GCAGAAGCTGAAAAGTTCAAGGC	94°C,45'', 55 °C,45'', 72 °C,45''
	HSP70as	CTTCTGCAGCAATCTTATCAGCAAA	
U6 snRNA (EHU43841)	EhU6 F	GGATCCACTTCGGTGGAAAT	94°C,45'', 62°C,45'', 72°C,30"
	EhU6 R	CTTCTCGTATGAGCGTGTCATC	
U2 snRNA (BK006130)	T7U2	GCATGCTAATACGACTTCTCGGCCTTTATG	94°C,45'', 60°C,45'', 72°C,45"
	hU2siR	TAACAGATCTTGTTTCCATGCACATCCTCG	
pEhExHA (Cysteine synthase)	CS+116	AGAATAGACTCTCATCAA	94°C,45'', 55°C,45'', 72°C,45"
	CS-103	GCTTTGTAACATCTTCTTGC	
pCR2.1	M13F	GTAAAACGACGGCCAGT	94°C,45'', 53°C,45'', 72°C,45"
	M13R	CAGGAAACAGCTATGACC	

Amplicon length
US = 510 bp<br>S = 374 bp
flicX13 = 116 bp
flicX13 = 79 bp<br>(using Rab2BSs)
Promoter = 215 bp
flicL12 = 100 bp
flicS14 ≈ 69 bp
flicCl2B ≈ 79 bp
Promoter = 341 bp
Promoter = 349 bp
flic670i1 = 110 bp
flic670i2 = 100 bp
circ670e2= 354 bp
Promoter = 125 bp
182 pb
flic510= 73 bp

[illegible]
Promoter = 347 bp
Promoter = 257 bp
178 pb
flic170= 113 bp
Promoter = 278 bp
Promoter = 455 bp
Promoter = 364 bp
Promoter = 321 bp
Promoter = 479 bp
Promoter = 354 bp
186 pb
Promoter = 283 bp
Promoter = 365 bp
Promoter = 392 bp
Promoter = 399 bp
121 bp
Promoter = 250 bp
Promoter =   250 bp
189 pb
Promoter = 250 bp

Promoter = 250 bp
190 pb
535 bp
4911 bp
204 bp
150 bp
92 bp
200 bp
-
-
